# A conversation with Zhijian (James) Chen

**DOI:** 10.1172/JCI189543

**Published:** 2025-01-02

**Authors:** Ushma S. Neill

Zhijian (James) Chen (University of Texas Southwestern and HHMI) has had a storied research career focused on the molecular mechanisms of innate immunity, the body’s immediate response to pathogens. For his discovery of the cGAS enzyme that senses foreign and self-DNA, Chen was awarded the 2024 Albert Lasker Basic Medical Research Award. Hear more from Chen (Figure 1) on the *JCI*’s website: https://www.jci.org/videos/cgms.

*JCI*: Where and how did you grow up?

Chen: I was born and raised in a small village in the southern part of China called Nando, which is in Anxi County, Fujian province. It’s a village surrounded by mountains that houses a few hundred people. We were annoyed by all the mountains back then because they kept us poor and isolated; now after living in Dallas for 30 years, I appreciate those mountains a lot more. We saw very few cars. Occasionally, there were some trucks driving through the village, and all the boys would come out and look at the trucks; the highest ambition for the boys in the village was to be a truck driver.

My father worked in the county government as a civil servant, but was stationed quite far away, so we didn’t see him often. My mother was an elementary school teacher. She had to teach in elementary school and then also do farm work and raise three kids, as I have 2 younger brothers. I was the oldest, so I did some work to help my mom. We didn’t have running water, so I had to get up early to get water from the wells. When I was about six years old, I started going to school, not as a student, but my mom wanted to keep me in the classroom to keep me out of trouble. I was born in 1966, the year when the Cultural Revolution started. That lasted for about 10 years; when I was in school, the whole education system was in repair mode. To a certain extent, I benefited in that I graduated from high school when I was 15.

*JCI*: What were your options for college and what did you want to study?

Chen: When I applied for college, I didn’t think about studying biology at all — I wanted to study math and physics. For whatever reason, I was accepted into a school that I didn’t apply to (Fujian Normal University) and into a major that I didn’t apply to (biology). I was very disappointed and so the first year in college, I was miserable. In my second year, I took a chemistry class with an inspirational teacher named Zhang Qichang. I found biochemistry very interesting, trying to understand life through the lens of chemistry.

I learned about the concept of graduate school and also learned that to get into graduate school, I needed to learn English. Before I graduated from college, I took some examinations and did quite well in English and biochemistry. Because I did quite well in those exams, I was selected for an overseas scholarship program. Before that, I never thought about going abroad for advanced study.

*JCI*: How is it that from a remote village in China, you picked the State University of New York in Buffalo for your PhD?

Chen: It just shows how ignorant I was at the time. I thought that to get to a PhD degree, I had to first get a master’s degree. I applied to a few MS programs in the US and got rejected by almost all of them. One day, I received a letter from SUNY Buffalo saying, “We are pleased to accept you into our graduate program, but this will be straight into a PhD program, not a master’s.” I thought this was amazing and that’s how I ended up in Buffalo.

*JCI*: How did you pick the lab of Cecile Pickart, studying ubiquitin?

Chen: She was a young assistant professor who I met when faculty introduced their research programs to incoming graduate students. I heard her presentation about the ubiquitin pathway and found the science to be beautiful. She was an amazing enzymologist, and I learned biochemistry and protein purification firsthand from her; she taught me side by side. Those lessons became very important for the rest of my career. I was fortunate to have a co-mentor, Ed Niles, who was a virologist. He taught me molecular cloning.

My PhD thesis consisted of discovering, purifying, and cloning a ubiquitin-conjugating enzyme called E2-25K. I identified its unique activity in making polyubiquitin chains. It was really a wonderful experience that has benefited me for the rest of my life. I also remember reading papers at that time by Bob Roeder from Rockefeller University. He was purifying transcription factors one after another, and I thought it was beautiful work. That inspired me, and I said, “This is what I want to do—purify proteins and identify their functions.”

*JCI*: You then moved to the Salk Institute to take up a postdoc with Inder Verma focusing on NF-κB.

Chen: I wanted to switch to another field and had become interested in transcription factors partly from reading those papers from Bob Roeder and others like Phil Sharp. Also, there was a lot of snow in Buffalo, and I wanted to be somewhere warm. I only applied to labs in California. and Inder was squarely in my focus as he was basically “Mr. c-fos.” After I arrived in the lab, Inder became interested in NF-κB, which had just been discovered by David Baltimore. Some of us newcomers started working on NF-κB.

*JCI*: Your postdoc was only about a year long, with a transition to Baxter Healthcare.

Chen: That was also not by design. I had a friend who applied for a position at Baxter, and he listed me as a reference. The headhunter called me to get the reference and then asked if I’d be interested in a position at Baxter too. I said no, but he convinced me to take a look. It was actually quite interesting and attractive. The position was focused on the first-generation CAR-T cells; at the time they were called “T-bodies.” They were ahead of their time, and it didn’t quite work. The job was also quite attractive because they offered me a fair bit more money than my postdoc did. I figured if it didn’t work out, I could always go back to another postdoc.

*JCI*: You ended up moving to ProScript for an additional couple of years working on Velcade. How did that transition happen?

Chen: I had kept in touch with Cecile Pickart, my PhD advisor. Cecile told me there was this startup company in Boston called Myogenics, that was to be the first company dedicated to the ubiquitin-proteasome pathway and they were looking for people who had experience in the ubiquitin-proteasome pathway. The company wanted to make proteasome inhibitors to treat cancer cachexia. Back then, there were very few people who knew anything about this pathway. I came for an interview; they offered me a position.

I spent half of my time helping to make a drug and developing assays for proteasome activity using ubiquitin conjugates. That work was quite meaningful because it led to the development of a proteasome inhibitor called MG341 that changed its name to PS341 when the company changed its name to ProScript. Later the company got bought by Leukocyte, and the compound changed its name to LS341and then the company was bought by Millennium Pharmaceuticals and changed its name to MN341. Eventually it went through clinical trials and became Velcade.

Then the other half of my time I was able to do basic research. The “other half of my time” meant weekends and late evenings, but during this time I was fortunate to collaborate with Tom Maniatis and others, dissecting the role of the ubiquitin pathway in NF-κB activation.

I showed that NF-κB activation involves ubiquitin-mediated degradation of an inhibitor called IκB. I made a very surprising observation that for IκB to be ubiquitinated, it must be phosphorylated. In order for IκB to be phosphorylated, it involves a ubiquitination event and that is required for this kinase activation. That was quite surprising because back then there were examples of phosphorylation leading to ubiquitination, but there was no precedent for ubiquitination regulating phosphorylation. Together with Tom, I published this in a paper in *Cell*.

This work showed that ubiquitin not only plays a role in protein degradation, but also in regulating signaling pathways. It also connected my research in ubiquitin and NF-κB, which became a central theme for my future work. I realized that to fully explore the implications of these findings, I needed to return to academia. Basic research like this wasn’t the primary focus of a biotech company, so I decided to apply for faculty positions.

*JCI*: I read that Eric Olson had just started a Department of Molecular Biology at UT Southwestern, and you were the very first recruit.

Chen: I applied for faculty positions and got a few offers, including from UT Southwestern. As you noted, Eric Olson had just started his department, and he was very effective at recruiting. When I was deciding among the offers, I got a phone call from Tom Maniatis who told me I ought to go to Dallas, saying it was the best place. There you had Mike Brown, Joe Goldstein, Al Gilman, Eric Olson, Steve McKnight, and others. A few minutes later, I got another phone call from Joe Goldstein. When Joe calls, nobody can resist; the rest is history.

*JCI*: You’ve demonstrated a continued devotion to Dallas. Is it true that you called your discovery mitochondrial antiviral signaling pathway (MAVS) after the Dallas Mavericks basketball team?

Chen: True story! After I started my lab at UT Southwestern, we continued to work on ubiquitin regulation of NF-κB. We made some solid discoveries, and I did enough to get myself tenure. But then I wanted to branch out a little bit, and at the time I had become interested in the signaling pathways triggered by viral infections.

Viral infections led to a very robust induction of interferon β. It requires NF-κB and other transcription factors, such as IRF3. We became interested in how RNA viruses induce type 1 interferons. We discovered a protein that is important for interferon induction by RNA viruses. We found that this protein is actually localized on the mitochondrial outer membrane; it is the first mitochondrial protein known to have a direct role in immunity. We wanted to have a name that started with M. At the time, the Dallas Mavericks were playing the Miami Heat in the NBA finals. We took inspiration.

I’ve had the opportunity to meet a few of the players. The Mavericks organize events for kids in the community, and I was invited to one of those events. I even got to shoot some hoops with the players, which was a lot of fun. I haven’t gotten my name on a jersey yet, though!

*JCI*: Your research trajectory turned toward DNA sensing. How did that become the question that you decided to focus on?

Chen: After we identified MAVS, there was increasing interest in how DNA, particularly microbial DNA, stimulates immune responses, especially the production of interferons. There were hints that MAVS might be involved in DNA sensing as well, but when we tested MAVS knockout cells, we found that they responded normally to DNA, which suggested there was another, distinct pathway for DNA sensing. At the time, the adapter protein called STING had been discovered by Glen Barber and Hongbing Shu and others. But it was clear that STING itself was not a DNA sensor.

We decided to take an unbiased approach to identify the DNA sensor using biochemistry. My postdoc, Josh Sun, worked on setting up a cell-free system to recapitulate DNA activation of interferon production. It took about two years to set up the assay and another year to purify the protein responsible for DNA sensing. That protein turned out to be an enzyme, which we named cGAS — cyclic GMP-AMP synthase. cGAS binds directly to DNA and becomes activated. Once activated, cGAS synthesizes a signaling molecule called cyclic GMP-AMP (cGAMP). This molecule then activates the STING pathway, leading to interferon production. The mechanism was elegant and simple.

*JCI*: If you could not have been a scientist or a physician, what else do you think could have motivated you over a career?

Chen: I cannot think of a better profession than being a scientist. It’s really a privilege to have the environment, the resources, the lab, and the trainees to make discoveries. Making discoveries is almost addictive. I do have a lot of interests outside science: I love music. I love history. One of my favorite things to do is to run or walk while listening to YouTube. When I was young, I didn’t really know anything; being in an isolated environment, going through a fast-track education, I really didn’t get a lot of education into history and music and other things. One of the nice things about being a scientist is that I get to travel quite a bit to attend conferences, to give talks. When I go to a new place in the world, I like to print out a Wikipedia page and really learn the history, the geography, anything interesting about a place. This was not imaginable when I was young.

## Figures and Tables

**Figure 1:**
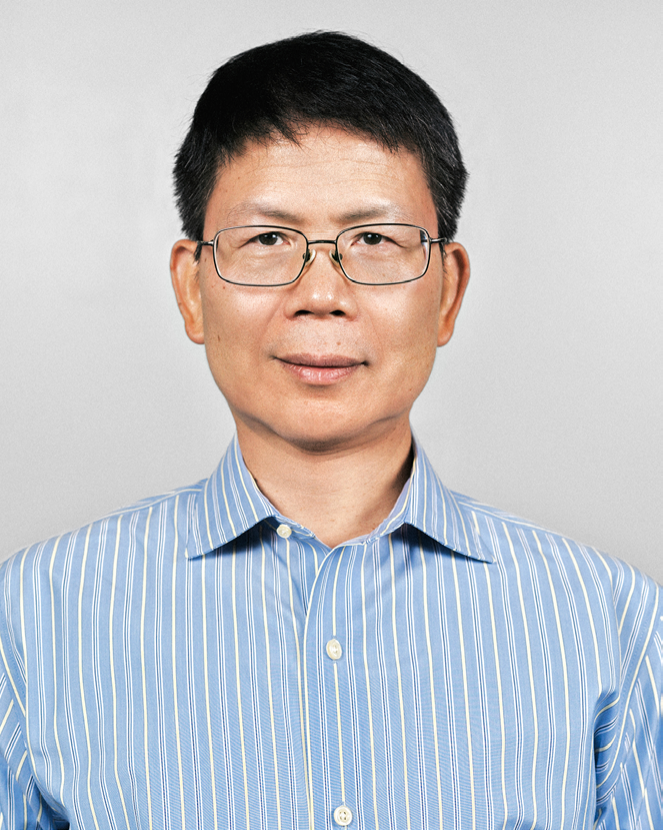
Zhijian James Chen on September 26, 2024 in NYC. Photo credit: Alexey Levchenko.

## Version 1 01/02/2025

Electronic publication

